# Relationship among school socioeconomic status, teacher-student relationship, and middle school students’ academic achievement in China: Using the multilevel mediation model

**DOI:** 10.1371/journal.pone.0213783

**Published:** 2019-03-20

**Authors:** Xin Xuan, Ye Xue, Cai Zhang, Yuhan Luo, Wen Jiang, Mengdi Qi, Yun Wang

**Affiliations:** 1 State Key Laboratory of Cognitive Neuroscience and Learning, Beijing Normal University, Beijing, China; 2 Collaborative Innovation Centre of Assessment toward Basic Education Quality, Beijing Normal University, Beijing, China; 3 Air China Limited Training & Education Department Southwest Branch, ShuangLiu International Airport, Chengdu, China; University of New England, AUSTRALIA

## Abstract

School socioeconomic status (SES) is studied primarily as a variable to explain academic achievement; however, few previous studies have investigated how SES can influence individual student’s academic achievement. The present study used a national representative sample of 10,784 grade 7 to 9 students (53.2% boys and 46.8% girls) in mainland China to examine the links between school SES and students’ math and Chinese achievements, including the math and Chinese teacher-student relationships as mediating factors. The parents provided family socioeconomic information and the students reported on their teacher-student relationships. Achievements in math and Chinese were assessed using standardized tests. Multilevel mediation analyses revealed that school SES was positively related to students’ math and Chinese achievements. Moreover, the link between school SES and students’ math achievement was partially mediated by students’ perception of the math teacher-student relationship. The Chinese teacher-student relationship had no mediating effect. This study indicated that school SES can influence individual student’s academic achievement via their perception of teacher-student relationship. The poverty and lack of resources is obvious, yet low SES schools could make efforts in improving teacher-student relationship’s quality to promote students’ academic performance. Meanwhile, low SES schools should receive more attention from policymakers to improve teaching quality and school climate. Furthermore, the study findings could be used for future research on the gap between low and high SES schools.

## Introduction

As a measurement of individual or collective social and economic status, socioeconomic status (SES) reflects existing or potential social resources such as wealth, power, and prestige [1]. School SES represents the average of each student’s family-based socioeconomic resources. It has attracted considerable attention since Coleman et al. [2] discovered the impact of ethnic and school socioeconomic composition on students’ academic achievement. Many studies have demonstrated that school SES is significantly related to students’ cognitive outcomes and academic achievement [3,4]. A meta-analysis including nearly 50 studies with samples of 6- to 18-year-old students indicated that both school and class SES have positive effects on students’ academic achievement in areas such as language, math, and science, with little difference in effect among the three subjects [5]. Furthermore, a study examining the changes in the relationship between school SES and 9-year-old students’ reading achievement in Sweden between 1991 and 2001 revealed that the positive effect of school SES on students’ reading achievement has been strengthened over time [6]. Palardy [7] conducted a longitudinal study with a nationally representative sample of American middle school students and examined the association between school SES and students’ achievement growth, revealing that high SES school students tend to have higher rate of achievement growth, even after controlling for an extensive set of students’ background characteristics and school inputs. Previous meta-analysis reviews and research from the Program for International Student Assessment (PISA), a global survey among OECD countries, found that school SES had a more significant effect on children’s academic achievement than that of family SES [4,8–11].

Despite growing interest in the relationship between school SES and students’ academic achievement, relatively few studies have explored the process factors through which school SES influences students’ academic achievement. Most researchers have considered the linking mechanism as a “black box” [5], and the majority of previous studies have been conducted in Western countries [12–14]. The present study, therefore, explored the relationship between school SES and students’ academic achievement and the underlying mechanism in China.

### Theoretical background

#### Effect of school SES

From a social science perspective, individuals’ surrounding social networks exercise a strong impact on their personal attitudes and behaviors [15]. For individual students, school SES reflects the social background of surrounding peers. Therefore, high family SES has a positive influence on a student’s academic performance; moreover, students belonging to the social network of high SES families are more likely to have better learning attitude and achievement, implying that a high school-wide SES is also positively related to students’ academic achievement; this effect is usually described as “peer effect” [16,17].

Roeser et al. [18] developed the “context-process-outcomes” model, which indicates that school context may influence students’ developmental outcomes through multiple process factors. These process factors as mediating roles transform the context inputs into outcomes, and being most effective in obtaining desired outcomes [19]. Specifically, the context includes factors such as achievement stimulants from higher administrative levels, school size, school category, and student composition; process characteristics, including school-level factors (e.g., educational leadership, disciplinary atmosphere), classroom-level factors (e.g., structured teaching, opportunity to learn, teacher-student relationships), and student-level factors (e.g., intrinsic motivation, academic engagement); and students’ developmental outcomes include intelligence and academic achievement [19,20]. Even though researchers have mentioned a series of process factors theoretically, many process factors have not been tested empirically. Furthermore, there is considerable uncertainty regarding the effectiveness of process factors, given the school context and student outcomes [19]. According to the “context-process-outcomes” model, school SES, as a context of students’ socioeconomic composition, may also influence students’ outcomes through some specific process factors. Teacher-student relationship, an aspect that reflects the connection between students and schools, has been demonstrated to be a significant factor affecting students’ outcomes [21–23]. However, further investigation is required on whether teacher-student relationship is an effective process characteristic between school SES and students’ outcomes.

### The mediating effect of teacher-student relationship

To elucidate the underlying mechanism in the relationship between school SES and students’ outcomes, researchers have begun to specify process variables. Liu et al. [24] examined whether school-level process factors mediate the relationship between school composition and student academic outcomes using data from 28 OECD countries in PISA 2003. They identified three meaningful mediators of school climate: disciplinary climates, students’ positive behavior, and student morale. According to previous research, high SES schools are characterized by higher level of collective teacher efficacy [25,26], which subsequently influences instructional strategies [27], classroom management [28], and teacher-student relationships [29]. Several researchers have proposed that high SES schools may get more support from parents [30]. Even though these studies have highlighted the influence of school SES on teaching, most of them have not examined the effect of school SES on teacher-student relationship; moreover, these studies have mainly focused on school-level process factors (e.g., disciplinary climates, collective teacher efficacy). To some extent, the effects of school-level processes were overlapping with those of school SES [31,32]. Therefore, it is necessary to test whether school SES directly impacts students’ perception of the teacher-student relationship at the individual level and whether it can further influence students’ academic performance.

When children enter school, teachers become important role models, surpassing even parents; they act as ad hoc attachment figures stemming from a sense of security [33]. A positive teacher-student relationship is related to students’ behavioral, cognitive, and social emotional development [34–36]. A meta-analysis of 99 studies that included students from preschool to high school revealed that both positive and negative teacher-student relationships were significantly related to students’ academic achievement [37].

Expectancy-value theory provides a theoretical foundation for the association between teacher-student relationship and students’ academic achievement [38,39]. Expectation refers to peoples’ beliefs regarding whether they can perform a task and the likely effects of various performances [40]. Value implies peoples’ criteria or frameworks against which the present experience can be tested [41]. In the school context, students who believe they can master their schoolwork typically have positive expectations for success; their expectations and value of the academic task contributes to their achievement. Wigfield et al. [38] proposed that students’ expectancies and values are influenced by the socializers with whom students have important relationships; in other words, teachers, as important socializers in school, can significantly impact students’ expectancies and values. Students who have a positive relationship with teachers are more likely to have positive expectances and values for success, further stimulating students’ study engagement and academic achievement. Thus, the expectancy-value theory implicates that teacher-student relationship, an important factor that influences students’ expectation and value of the school task, can greatly impact students’ academic performance.

Many studies have focused on how a general teacher-student relationship influences students’ outcomes [42,43]. In these studies, teacher-student relationship was assessed by students reporting their relationship quality with most of their teachers [44] or by the teacher who spends the longest time with students reporting his or her relationship quality with students [45]. PISA suggested focusing on “domain-specific” teacher-student relationship to describe how it influences students’ outcomes in specific classes [20]. The effect sizes of teacher-student relationships on different curriculums may vary in degree. Therefore, they asked students to report the relationship quality with mathematics, language and science teachers separately. The present study, then, examined the mediating effect of students’ perceptions of relationship quality with math and Chinese teachers on the association of school SES and student math and Chinese achievement, respectively.

In summary, the present study had two goals. The first was to examine the effect of school SES on math and Chinese performance among grades 7–9 students in mainland China, as middle school students entering puberty are more likely to be affected by their school’s unique environment [37]. The second goal was to examine the math and Chinese teacher-student relationship as potential process factors of the relationships between school SES (context) and students’ math and Chinese achievements (outcomes).

The present study tested two hypotheses regarding the relationship between school SES and students’ academic achievements:

**H1** School SES is positively related to middle school students’ math and Chinese achievements.

**H2** The math and Chinese teacher-student relationship are mediating factors between school SES and students’ math and Chinese achievements.

## Methods

### Ethics statement

The study was approved by the Institutional Review Board (IRB) of the State Key Laboratory of Cognitive Neuroscience and Learning at Beijing Normal University.

### Participants

The data used in this study were drawn from the National Children’s Study of China (NCSC), collected data from a nationally representative school-based sample of primary- and middle school students from 31 provinces in mainland China in 2009. A multistage, stratified, and unequal probability sample design was used to choose the final sample.

For this study, data were extracted from the academic achievement database of the NCSC. The sample consisted of 10,784 participants in grades 7–9 from 199 schools. The mean age was 14.52 years (SD = 1.11), and the sample contained 53.2% boys and 46.8% girls. Of these, 3609 were in grade 7 (33.5%), 3605 were in grade 8 (33.4%), and 3570 were in grade 9 (33.1%). Of the total schools, 71.9% were located in cities and 28.1% were located in rural areas; 95% were public schools and 5% were private schools. The number of students in each school ranged between 237 and 8170.

### Measures

#### School SES

School SES was the average of students’ family SES, which included indexes of parents’ highest education level and household income. Only one of the student’s parents reported, choosing their own and their spouse’s education level on a 13-category scale, from 1 = did not go to school; 2 = elementary school degree; 3 = middle school degree … 10 = bachelor’s degree; 11 = master’s degree or above; 12 = other; 13 = unknown. The rate of participants who chose options 12 and 13 education levels was less than 0.01%; therefore, these data were considered missing data. Annual household income was also reported by one of the student’s parents by asking the following: “What was the total income of all your family members from all sources (e.g., salaries, bonuses, and subsidies) after taxes in 2008?” Participants were instructed to select one of the following categories: 1 = less than RMB3,000; 2 = RMB3,001–RMB6,000; 3 = RMB6,001–RMB10,000 … 9 = more than RMB200,001. A principal component analysis (PCA) was conducted to create family SES [46].

#### Math and Chinese tests

Math and Chinese tests were developed by the NCSC researchers on the basis of curriculum standards and relevant research within China and abroad [47]. Each of the tests included three parallel testing papers (A, B, C) for a total testing time of 60 minutes.

The math test contained algebra and equations, spatial queries and geometry, statistics and probability, and practical application, to discover each student’s knowledge of the four levels: knowing facts, applying rules, mathematical reasoning, and innovative problem solving. Cronbach’s alpha for the three (A, B, C) test papers was 0.89, 0.90, and 0.90, respectively. The NCSC researchers chose semester math examinations of schools that used the same test paper as the criterion of the math test in this study. The criterion validity was greater than 0.8.

The Chinese test included language accumulation and reading, the weight of two parts was a ratio of 4:6. The language accumulation part focused on abilities of knowing, understanding, and applying Chinese cultural knowledge, while the reading part focused on acquisition ability, interpretation, and commenting. Cronbach’s alpha of the three (A, B, C) test papers was 0.81, 0.82, and 0.81, respectively. The NCSC researchers also chose semester Chinese examinations of the schools that used the same test paper as the criterion of the Chinese test in this study. The criterion validity was greater than 0.6.

#### Teacher-student relationship

The measures of math and Chinese teacher-student relationships were also developed by the NCSC [48] and each scale included four items. The items of math teacher-student relationship scale were, for example, “Math teacher encourages me to learn math”, “I get along well with the math teacher”, “The math teacher is very concerned about my math study” and “When I have difficulties in math learning, the math teacher helps me proactively.” Items on Chinese teacher-student relationship were similar to those items on the math teacher-student relationship scale. The responses were provided on a four-point, Likert-type scale, ranging from 1 = *complete disagreement* to 4 = *complete agreement*. All four items were summed to calculate the average score of the perceived math or Chinese teacher-student relationships; a higher score reflected a better relationship. Cronbach’s alpha of the math and Chinese teacher-student relationship scale was 0.83 and 0.80, respectively. Confirmatory factor analysis indicated reasonable construct validity of math teacher-student relationship scale (χ^2^ = 519.72, df = 2, CFI = 0.98, TLI = 0.96, RMSEA = 0.10) and Chinese teacher-student relationship scale (χ^2^ = 517.50, df = 2, CFI = 0.99, TLI = 0.96, RMSEA = 0.09).

### Covariates

For this study, student-level covariates included grade (7 = seventh grade, 8 = eighth grade, 9 = ninth grade), gender (1 = boy, 2 = girl), and family SES. Researchers have found girls were more likely to score lower than boys in mathematics, and the gap between girls and boys varied among countries [49].

School-level covariates were reported by the president of each school and included school location (1 = urban, 2 = rural), school type (1 = public, 2 = private), and school size. Previous studies have shown these three factors are related to students’ achievements. For example, Lee et al. [50] found that in the U.S., rural school students had lower math scores in 1992, but by 1996, they outperformed their non-rural counterparts. Cadigan et al. [51] used the data from PISA and revealed that private school students outperformed their public school peers in Canada. Furthermore, students in small schools tend to have higher achievement in Western countries [52,53]; however, researchers reported that school size was positively related with students’ science achievement in Hong Kong [54]. Nevertheless, the existing research findings are inconclusive and little is known about Chinese students. Therefore, these variables were regarded as covariates in this study.

#### Statistical analyses

Data were analyzed using the hierarchical linear model (HLM) 6.08 (Scientific Software International, Skokie, Il), because HLM can appropriately address the hierarchically nested design of this study [55]. In the study data set, students as individual-level units were nested within a group-level unit of their particular school [56].

We initially computed the intra-class correlation coefficient (ICC) for the outcome and mediator variables from the unconditional models. The ICC reflects that the variance of dependent variable can be explained by group-level properties [55,57]. Furthermore, if the ICC value exceeds the 0.05 criterion, it implies a significant variance of that dependent variable among groups [58], thereby necessitating a hierarchical linear analysis. In this study, math and Chinese were entered into the HLM analysis as dependent variables, with no predictors in the models, and the results indicated significant variances of math (ICC = 0.26) and Chinese (ICC = 0.19) among the schools. The results of math teacher-student relationship (ICC = 0.08) and Chinese teacher-student relationship (ICC = 0.08) were also significant. Therefore, in the analysis, school-level independent variables were entered into level-2 and student-level independent variables were entered into level-1 analysis.

The continuous variables, including the dependent variables, were standardized using Z scores across all of the schools included in the study. This method is similar to the grand-mean centering method [59] suggested by statistical methodologists [60]. Dummy variables such as gender, grade, school location, and school type were uncentered. Listwise deletion was also used in the HLM analyses because a low rate (0.1%–3.4%) of participants had missing data in student-level variables; two schools had missing data on the variable of school size.

Multilevel mediation was then used among students’ math and Chinese achievements separately as follows. First, the independent variable (school SES) must be related to the dependent variables (math or Chinese) after controlling for the student level (grade, gender, family SES) and school level (school location, school type, school size) covariates: coefficient *c* in Eq 1.

$$\begin{array}{l} Level\;1:\;math/Chinese\;achievement_{ij}=\beta_{0j}+\beta_{1j}{\left(grade\right)}_{ij}+\beta_{2j}{\left(gender\right)}_{ij}+\beta_{3j}{\left(family\;SES\right)}_{ij}+\gamma_{ij}\\ Level\;2:\;\beta_{0j}=\gamma_{00}+\gamma_{01}{\left(school\;location\right)}_{j}+\gamma_{02}{\left(school\;type\right)}_{j+}\gamma_{03}{\left(school\;size\right)}_{j}+c/\;\gamma_{04}{\left(school\;SES\right)}_{j}+\mu_{0j} \end{array}$$ (1)

Second, the independent variable (school SES) must correlate with the mediator (math/Chinese teacher-student relationship) after controlling for covariates: coefficient *a* in Eq 2.

$$\begin{array}{l} Level\;1:\;math/Chinese\;teacher‑student\;relationship_{ij}=\beta_{0j}+\beta_{1j}{\left(grade\right)}_{ij}+\beta_{2j}{\left(gender\right)}_{ij}+\beta_{3j}{\left(family\;SES\right)}_{ij+}\gamma_{ij}\\ Level\;2:\;\beta_{0j}=\gamma_{00}+\gamma_{01}{\left(school\;location\right)}_{j}+\gamma_{02}{\left(school\;type\right)}_{j}+\gamma_{03}{\left(school\;size\right)}_{j}+a/\;\gamma_{04}{\left(school\;SES\right)}_{j}+\mu_{0j} \end{array}$$ (2)

Third, the mediator must be associated with the dependent variable (math/Chinese achievement) when the independent variable (school SES) was controlled for: coefficient *b* in Eq 3. The association between school SES and math/Chinese achievement was presented by coefficient *c’*.

$$\begin{array}{l} Level\;1:\;math/Chinese\;achievement_{ij}=\beta_{0j}+\beta_{1j}{\left(grade\right)}_{ij}+\beta_{2j}{\left(gender\right)}_{ij}+\beta_{3j}{\left(family\;SES\right)}_{ij}+b/\beta_{4j}{\left(math/Chinese\;teacher-student\;relationship\right)}_{ij}+\gamma_{ij}\\ Level\;2:\;\beta_{0j}=\gamma_{00}+\gamma_{01}{\left(school\;location\right)}_{j}+\gamma_{02}{\left(school\;type\right)}_{j+}\gamma_{03}{\left(school\;size\right)}_{j}+c’/\;\gamma_{04}{\left(school\;SES\right)}_{j}+\mu_{0j}\\ \;\beta_{1j}=\gamma_{10} \end{array}$$ (3)

The multiplication of paths *a* and *b* yielded the indirect effect of school SES on students’ math/Chinese achievement. Partial mediation occurred when the path from school SES to students’ math/Chinese achievement was reduced, but was still significant with the mediator (math/Chinese teacher-student relationship) in the model. Complete mediation occurred when the path from school SES to students’ math/Chinese achievement was no longer significant as the presence of the mediator.

## Results

### Preliminary analysis

The results of the PCA to establish family SES showed that there was a reasonable principal component to indicate family SES and the factor structure fit well. The result of scree plot highlighted two factors. The eigenvalue of the two factors were 1.38 and 0.62, respectively, and the scree curve was flat from the second factor. According to the standard of eigenvectors over 1 and scree plot [61,62] we derived one principal component from the construct of parents’ highest education level and annual household income as the family SES. The communality value of parents’ highest education level and household income both was 0.69. The total variance explained by the principal component was 69.02%. Then we averaged students’ family SES from the same school to create school SES.

### Descriptive and correlation analysis

Table 1 presents the descriptive and correlational results of all variables in the research; it is evident that school SES and family SES are positively correlated. Among the student-level variables, grade and family SES were positively related with students’ math and Chinese achievements. Both higher grade and higher family SES scored better. Gender was only related with Chinese achievement; girls tended to have higher Chinese achievement. Among the school-level variables, school location, school type, school size, and school SES were all significantly related to students’ math and Chinese achievements. School location, school type, and school size were also significantly correlated with school SES. Urban schools were likely to have higher SES and performed marginally better than rural schools. Private schools also had higher SES and performed better than public schools. Furthermore, higher SES schools were larger and performed better.

**Table 1:** Descriptive and correlational statistics for student and school variables.

		1	2	3	4	5	6	7	8	9	10	11
*Correlations*												
1	Gender	-										
2	Grade	0.01	-									
3	Family SES	0.01	0.02*	-								
4	Math teacher-student relationship	-0.01	-0.07**	0.10**	-							
5	Chinese teacher-student relationship	0.02*	-0.08**	0.06**	0.53**	-						
6	School location	-0.03	0.01	-0.41**	-0.10**	-0.10**	-					
7	School type	-0.04**	-0.01	0.12***	0.08***	0.07***	-0.01	-				
8	School size	0.02	-0.01	0.23**	0.02	0.06**	-0.22**	0.06*	-			
9	School SES	0.01	-0.01	0.64**	0.13**	0.09**	-0.64**	0.19***	0.35**	-		
10	Math score	-0.02	0.45**	0.30**	0.17**	0.03*	-0.15**	0.07*	0.16**	0.35**	-	
11	Chinese score	0.11**	0.31**	0.30**	0.10**	0.03*	-0.14**	0.04*	0.17**	0.32**	0.69**	-
*Descriptive statistics*												
	Mean	-	-	0	1.78	1.82	-	-	1609.41	0.01	510.88	508.48
	SD	-	-	1	0.74	0.70	-	-	123.06	0.64	100.20	99.34
	Min	-	-	-1.64	1	1	-	-	237	-1.03	226.04	178.55
	Max	-	-	3.71	4	4	-	-	8170	2.42	782.41	815.27

Note. Gender: 1 = male, 2 = female. Grade: 7 = 7^th^ grade, 8 = 8^th^ grade, 9 = 9^th^ grade. School location: 1 = urban, 2 = rural. School type: 1 = public, 2 = private.

* *p* < 0.05.

** *p* < 0.01.

***p < 0.001

### Mediation analyses

#### Math

As discussed above, the direct effect of school SES (independent variable) on students’ math achievement (dependent variable) and math teacher-student relationship (mediate variable) was investigated first, after controlling for all covariates. As shown in Table 2 (step 1), grade (*β* = 0.45, *p* < 0.001), gender (*β* = -0.03, *p* < 0.05), family SES (*β* = 0.12, *p* < 0.001), and school location (*β* = -0.11, *p* < 0.001) had significant direct effects on math achievement, suggesting that male students in higher grades with higher family SES in urban schools reported higher math achievement. School SES was positively related to students’ math achievement (*β* = 0.32, *p* < 0.001). Furthermore, according to Table 2 (step 2), grade (*β* = -0.07, *p* < 0.001), family SES (*β* = 0.03, *p* < 0.05) and school size (*β* = -0.07, *p* < 0.01) were significantly related with math teacher-student relationship, wherein lower grade students from small schools with higher family SES reported better relationships with math teachers. There was a significant effect of school SES on math teacher-student relationship (*β* = 0.11, *p* < 0.01), implying that schools with higher SES were associated with better student-teacher relationships.

**Table 2:** Mediation analyses: Association between school SES and students’ math achievement through math teacher-student relationship.

	Step1		Step 2		Step 3	
	math achievements (ICC = 25.92%)		math teacher-student relationship (ICC = 8.04%)		math achievement (ICC = 25.92%)	
	*Β*	SE	*Β*	SE	*β*	SE
Intercept	-0.01	0.02	-0.01	0.02	-0.01	0.02
Level 1 covariates						
Grade	0.45***	0.02	-0.07***	0.01	0.46***	0.01
Gender	-0.03*	0.01	-0.02	0.01	-0.02*	0.01
Family SES	0.12***	0.01	0.03*	0.01	0.12**	0.01
Level 2 covariates						
School location	-0.11**	0.03	-0.04	0.03	-0.11**	0.03
School type	-0.01	0.03	-0.01	0.03	-0.01	0.03
School size	-0.01	0.02	-0.07**	0.02	0.05	0.02
Level 2 independent						
School SES	0.32***	0.03	0.11**	0.03	0.30***	0.03
Level 1 mediator						
Math teacher-student relationship					0.15***	0.01
Proportion reduction in error at level 1	28.0%		0.6%		30.6%	
Proportion reduction in error at level 2	52.8%		18.0%		55.4%	

Note.

* p < 0.05.

** p < 0.01.

***p < 0.001

**Fig 1:**
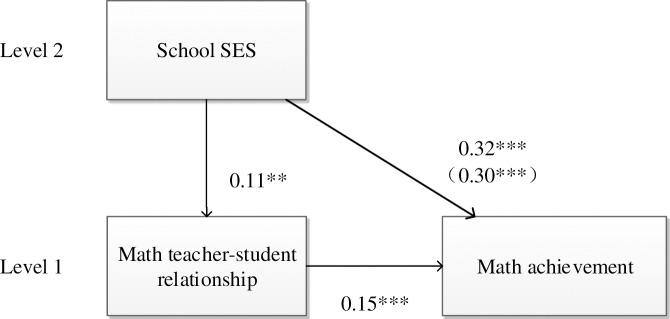
Mediation model for math.

We then established the effect of math teacher-student relationship on students’ math achievement, after controlling for the independent variable (school SES) and other covariates. The results revealed that math teacher-student relationship was significantly associated with students’ math achievement (*β* = 0.15, *p* < 0.001), when the school SES variable was controlled for (Table 2, step 3). The relationship between school SES and students’ math achievement was still significant (*β* = 0.30, *p* < 0.001), when math teacher-student relationship was considered, indicating a partial mediation of math teacher-student relationship. A detailed model is presented in Fig 1.

#### Chinese

**Fig 2:**
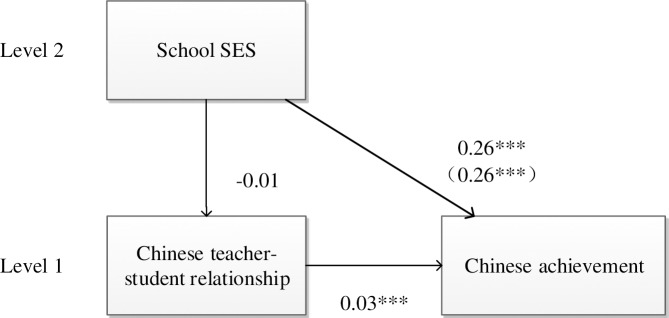
Mediation model for Chinese.

As evident in Table 3 (step 1), grade (*β* = 0.30, *p* < 0.001), gender (*β* = 0.10, *p* < 0.001), family SES (*β* = 0.16, *p* < 0.001), school location (*β* = -0.11, *p* < 0.001), and school size (*β* = 0.06, *p* < 0.05) had significant direct effects on Chinese achievement; thus, higher grade female students with higher family SES in a large, urban school reported higher Chinese achievement. School SES was also positively related to students’ Chinese achievement (*β* = 0.26, *p* < 0.001). Furthermore, according to Table 3 (step 2), grade (*β* = 0.08, *p* < 0.01), school location (*β* = -0.07, *p* < 0.05), and school size (*β* = -0.10, *p* < 0.01) were significantly associated with Chinese teacher-student relationship, implying that higher grade students in large, urban schools had better Chinese teacher-student relationship. School SES was not related with Chinese teacher-student relationship (*β* = -0.01, *p* > 0.05), meaning that Chinese teacher-student relationship was not a significant mediator between school SES and students’ Chinese achievement (Table 3, step 2). While the mediating effect of Chinese teacher-student relationships was not significant, the direct effect of this relationship on students’ Chinese achievement was still investigated. As shown in Table 3 (step 3), school SES (*β* = 0.26, *p* < 0.001) and Chinese teacher-student relationship (*β* = 0.03, *p* < 0.01) were positively related with students’ Chinese achievement. A detailed model is presented in Fig 2.

**Table 3:** Mediation analyses: Association between school SES and students’ Chinese achievement through Chinese teacher-student relationship.

	Step1		Step 2		Step 3	
	Chinese achievements (ICC = 18.76%)		Chinese teacher-student relationship (ICC = 8.33%)		Chinese achievement (ICC = 18.76%)	
	*Β*	SE	*Β*	SE	*β*	SE
Intercept	-0.01	0.02	0.01	0.02	-0.01	0.02
Level 1 covariates						
Grade	0.30***	0.02	0.08**	0.01	0.31***	0.01
Gender	0.10***	0.01	-0.02	0.01	0.10***	0.01
Family SES	0.16***	0.01	-0.01	0.01	0.15***	0.01
Level 2 covariates						
School location	-0.11**	0.03	-0.07*	0.03	-0.12***	.03
School type	-0.02	0.03	0.01	0.02	-0.02	0.03
School size	0.06*	0.02	-0.10**	0.02	0.06	0.02
Level 2 independent						
School SES	0.26***	0.03	-0.01	0.03	0.26***	0.03
Level 1 mediator						
Chinese teacher-student relationship					0.03**	0.01
Proportion reduction in error at level 1	15.1%		0.7%		15.2%	
Proportion reduction in error at level 2	51.0%		12.0%		51.0%	

Note.

* p < 0.05.

** p < 0.01.

***p < 0.001

## Discussion

Using a multilevel mediation approach, the current study examined the mediating effect of teacher-student relationships between school SES and students’ academic achievement in math and Chinese. The findings indicated that school SES significantly predicted middle school students’ math and Chinese performance. The math teacher-student relationship partially mediated the relationship between school SES and math achievement, however, the mediating effect of Chinese teacher-student relationship was not significant.

### Association between school SES and students’ academic achievement

Students’ family SES, grade, gender, and relevant school characteristics were controlled for in the current study and the results were consistent with those of the previous studies [6,11,63], revealing that students achieved higher math and Chinese scores in high SES schools. In addition, school SES was correlated with family SES, suggesting that most high SES families were grouped into high SES schools and a majority of low SES families were grouped into low SES schools. Furthermore, large, private urban schools were likely to have higher SES. According to the “peer effect” [16,17], students in high SES schools, surrounded by peers from high SES families, are influenced by their peers to engage in study and achieve high scores. Therefore, students in high SES schools, regardless of high or low family SES, are more likely to gain higher academic achievement. Conversely, students surrounded by low SES peers may be influenced by their negative learning attitude and behavior to gain poor academic performance, which needs further investigation. Furthermore, the average education expenditure of urban middle school students is higher than that of rural middle school students, and the gap is widening from 2006 to 2015 in China [64,65]. Meanwhile, the level of teaching resources in rural areas also remains relatively weak [66]. Because most of low SES schools are distributed in rural areas, these schools are more likely to have less financial funding and less high-quality teachers. Thus, students in low SES schools cannot receive sufficient support and gain poor academic performance.

### The mediation of teacher-student relationship between school SES and students’ academic achievement

The present study confirmed that the math teacher-student relationship was a significant process factor between school SES and students’ math achievement, which was consistent with the model of “context-process-outcomes” [18] and “expectancy-value-theory” [38,39]. School SES as a contextual factor can affect students’ perception of teacher-student relationship in math and further influence students’ math achievement. The correlation result in this study showed that school SES was significantly correlated with students’ perception of relationship with math teacher. Students in high SES schools perceived better relationship with math teachers than their counterparts in low SES schools. According to previous studies, teachers in low SES schools have reported that their students are less teachable [67,68] and that they have a lower level of trust on their students [69,70]. Therefore, the teacher-student relationship quality in low SES can be considered worse, which negatively impacts students’ achievement [71,72].

This study, however, didn’t find significant mediating effect of the Chinese teacher-student relationship; in addition, high school SES did not predict students’ perception of the Chinese teacher-student relationship. There was no difference of Chinese teacher-student relationship quality between high and low SES schools. This difference between math and Chinese teacher-student relationships may be due to the different characteristics between these two subjects; for example, junior high school math learning may be more dependent on teacher intervention than the same level of Chinese learning. Middle school mathematics in China is characterized by challenging problem solving and sequential development of content without repetition, the intensity of which was found to be higher than that in American middle school mathematics curriculum [73]. Furthermore, Chinese culture attaches more importance to the study of mathematics [73,74]. Thus, teachers in high SES schools may provide more support for students in math problem solving and maintain a good relationship with students. Chinese learning involves students’ learning habits, accumulation, and reading activities outside class, with issues that can be frequently solved by the students themselves. The contribution of Chinese teachers’ instruction to students’ achievement in schools with different SES may be equally. Thus, students’ perceived relationship with Chinese teacher was not significantly different among schools. The reason for students in high SES schools to achieve high scores in Chinese examinations may be that families and schools provide more books and reading activities for students to obtain Chinese knowledge. Even though school SES was not significantly related with Chinese teacher-student relationship, teacher-student relationship was positively correlated with students’ Chinese achievement. Thus, good Chinese teacher-student relationship could also improve students’ Chinese achievement. However, researchers must further explore significant process factors between school SES and students’ Chinese achievement.

### Limitations and future directions

There are several limitations of this study that need to be considered. First, the data used in this study came from large, cross-sectional survey research, so the results are limited to drawing causal conclusions. To reach causal conclusions, independent variables should precede dependent variables in time [75]. Therefore, future research could investigate such causal relationships by designing a longitudinal study. Second, listwise deletion was used to deal with missing data in this study, given the low missing rate, it has some disadvantages; primarily, listwise deletion results in some loss of power and biases because of unused partial data, especially when the missing rate is high [76,77]. Future studies could use other imputation methods (e.g., multiple imputation) to handle missing data. Third, this study did not control for students’ initial achievement level, learning motivation, and other factors that may impact their academic achievement, which may overestimate the impact of school SES on students’ achievements. Finally, this study was only concerned with the impact of school SES on students’ academic achievement, and that a high school SES would have a positive effect on academic achievement in students from low SES families. However, some previous studies have presented contradicting findings wherein an increased proportion of students from high SES families has resulted in students from low SES families to exhibit slower learning speeds in math and science [78]. Future research could specify how school SES influences low SES students and expand these studies to discuss the impact of school SES on students’ psychosocial development.

### Implications and conclusion

The present study tested the association between school SES and students’ academic achievement and examined the mediating role of teacher-student relationship on this association. There are theoretical and practical implications of the findings. First, the sample of this study was nationally representative of middle school students from mainland China, and the findings further verify the “context-process-outcomes” model [18]. We found school SES (context) through math teacher-student relationship (process) influence students’ math achievement (outcome). This proves the impact of school environment on students’ academic achievement in Chinese schools. Second, school SES was positively related with middle school students’ math and Chinese achievement after controlling family SES. This finding suggests that government and other educational practitioners should not have low SES students grouped together to form low SES schools, as it can aggravate low family SES students’ negative developmental outcomes. Third, different results regarding teacher-student relationships in math and Chinese subjects indicates that there was a difference in the process factors for these different subjects; therefore, the government and schools should work to improve the quality of process factors according to the characteristics of specific subjects. Finally, our study confirms the mediating effect of math teacher-student relationship on the association between school SES and students’ math achievement, implying that schools with low SES have not only substandard material conditions but also poor interpersonal climate for student learning. Although we are unable to change the effect of school SES, which could be treated as a distal factor, we could make efforts in changing the proximal factor (teacher-student relationship) to promote students’ academic performance. Therefore, families and schools should improve students’ development jointly: school administrators should create a supportive environment for a positive teacher-student relationship climate; teachers should pay more attention to students’ real needs and establish a good relationship with students; and parents should be more actively involved in school activities to strengthen communication with teachers.
